# Expectant Management of Monochorionic-Triamniotic Triplets Complicated by Selective In Utero Growth Restriction: Report of 2 Cases

**DOI:** 10.1155/2020/2979261

**Published:** 2020-10-03

**Authors:** Laurence Carmant, Sandrine Wavrant, Elisabeth Codsi

**Affiliations:** Department of Obstetrics and Gynecology, Division of Maternal Fetal Medicine, Sainte-Justine Mother and Child University Hospital, 3175 Chemin de la Côte Sainte Catherine, Montreal, QC, Canada H3T 1C5

## Abstract

The optimal management of monochorionic-triamniotic (MCTA) triplet pregnancies is not clearly established, and there is no literature to guide management of MCTA complicated with selective intrauterine growth restriction (sIUGR). This gap in knowledge and the concern for higher risk of severe complications have led some medical societies to recommend selective termination of nontrichorionic triplet pregnancies. We sought to report the favourable outcomes of two MCTA complicated by sIUGR expectantly managed at Sainte-Justine Hospital, Montreal, Canada. The first case is of a 42-year-old woman with spontaneous MCTA triplets diagnosed at 18 weeks with type II sIUGR who opted for expectant management. The second patient was a 22-year-old woman with a spontaneous MCTA triplet pregnancy diagnosed at 18 weeks with type III sIUGR. Our experience shows that close serial ultrasounds could potentially allow physicians to foresee fetal deterioration. In our opinion, expectant management should be considered as a management option for MCTA complicated by sIUGR.

## 1. Introduction

In North America, triplets and higher-order multifetal gestations account for approximately 153.4 per 100 000 live births. The increasing incidence of multifetal gestations reflects older maternal age at conception and the growing use of assisted reproductive technologies. These pregnancies are associated with increased fetal and neonatal morbidity and mortality mainly due to prematurity [1]. Literature has also shown that perinatal morbidity and mortality in multifetal gestations are strongly linked to chorionicity [2, 3]. Indeed, dichorionic-triamniotic (DCTA) and monochorionic-triamniotic (MCTA) triplet pregnancies are at higher risk of complications compared to trichorionic-triamniotic (TCTA) pregnancies [4, 5]. This has led the North American Fetal Therapy Network (NAFTNet) to state that because of the higher complication rates, parents of nontrichorionic triplets should be counseled about the option of selective fetal reduction of the monochorionic pair [6]. However, when faced with MCTA triplets, the optimal management is less clear. Moreover, there is no literature to guide management of MCTA complicated with selective intrauterine growth restriction (sIUGR). Therefore, we sought to report the outcomes of two MCTA complicated by sIUGR that were expectantly managed at the Sainte-Justine Mother and Child University Hospital.

## 2. Case Presentation #1

A 42-year-old woman, gravida 3 para 1 avorta 1, was diagnosed with spontaneous MCTA triplets during nuchal translucency (NT) ultrasound. All fetuses had a normal NT. Selective fetal reduction was discussed, but the patient opted for expectant management. Low-dose aspirin was initiated. All her first trimester laboratory tests were normal. She proceeded with amniocentesis at 15 weeks. All 3 karyotypes were normal. At 18 weeks, morphology ultrasound was normal but showed sIUGR of fetus B, for which she was referred to our center. A detailed level II ultrasound confirmed the diagnosis of type II sIUGR in triplet B. Indeed, triplet B estimated fetal weight was less than the fifth percentile, there was intertriplet discordance of more than twenty-five percent and its umbilical artery (UA) Doppler showed absent diastolic flow. However, its middle cerebral artery (MCA) and ductus venosus (DV) Dopplers were both normal. Twin B had a velamentous cord insertion. There was no sign of twin-twin transfusion syndrome (TTTS) or twin anemia polycythemia sequence (TAPS). Selective fetal reduction using radiofrequency ablation was again discussed with the new findings; however, the patient opted for expectant management. She underwent weekly ultrasound. At 22 weeks, she underwent fetal echocardiography, which only showed a mild pulmonary valve dysplasia on fetus A. Triplet B followed its <5^th^ percentile growth curve and Dopplers remained stable from week to week, and the patient underwent caesarean section at 32 weeks after a course of betamethasone. All three babies had a normal pH and APGAR at birth and now have normal development at 1 year of age. The patient was discharged from the hospital postpartum day 5 because she suffered a nonsevere postpartum preeclampsia. Her blood pressure and laboratory testing were back to normal at her 6 weeks' postpartum follow-up visit.

## 3. Case Presentation #2

A 22-year-old woman primigravida with a spontaneous MCTA triplet pregnancy was referred to our center for a suspicion of sIUGR of triplet B at 18 weeks. A detailed level II ultrasound confirmed the diagnosis of type III sIUGR in triplet B. Indeed, triplet B estimated fetal weight was less than the fifth percentile, there was intertriplet discordance of more than twenty-five percent, and it showed intermittently absent/reverse diastolic flow in the umbilical artery of twin B. Middle cerebral artery and ductus venosus Dopplers were both normal. Twin B had a velamentous cord insertion. Selective fetal reduction was discussed, but the patient opted for expectant management. She underwent weekly ultrasound. Triplet B followed its <3^rd^ percentile growth curve. At 27 weeks, the DV Doppler of triplet B showed a pulsatility index above the 95^th^ percentile. Occasional spontaneous heart rate decelerations were also noted. The patient was immediately hospitalised and underwent a C-section after a course of betamethasone and four hours of magnesium sulfate. All three babies had normal pH and APGAR at birth and have normal development at 1 year of age.

## 4. Discussion

Monochorionic-triamniotic triplets are inherently at higher risk of adverse pregnancy outcomes than higher chorionic multiple gestation pregnancy. With added complications such as TTTS, TAPS, or sIUGR, it seems reasonable to opt for selective fetal reduction as it can correct both the complication and the burden of having a higher order multiple fetal gestation. However, fetal therapy is not without significant risk for the pregnancy and the neurological prognosis of the remaining cotriplet. Van Schoubroeck and colleagues published a case series in which 3 MCTA triplets underwent cord occlusion for TRAP, 1 for fetal anomaly and 1 laser photocoagulation for TTTS. They reported outcomes similar to those described in larger series of twin pregnancies. They therefore concluded that laser photocoagulation was preferable to expectant management in MCTA complicated with TTTS. Moreover, they deemed second-trimester cord occlusion a reasonable option for TRAP or major discordant anomaly [7]. However, they did not have cases of MCTA complicated by sIUGR. NAFTNet states in their 2015 consensus that for type II or III sIUGR, selective feticide can be considered but laser photocoagulation should remain experimental [3]. Gratacos and colleagues also prefer cord occlusion to laser photocoagulation for type II and III sIUGR when fetal therapy is the chosen option [8].

Our experience with these MCTA triplets shows that we can achieve good outcomes with expectant management. Close serial ultrasounds allow the physician to foresee fetal deterioration even in more unpredictable sIUGR such as type III. As shown in the literature, abnormal DV Doppler is associated with adverse outcomes in monochorionic-diamniotic twins, which may be extrapolated to MCTA [9]. In our 2 cases, DV Dopplers were normal, suggesting that the findings in MCDA could potentially be extrapolated to MCTA pregnancies. It is important to emphasize that these patients were managed in a quaternary care hospital with optimal resources. The outpatient follow-up protocol in our institution consisted of weekly ultrasound for UA/MCA/DV Doppler and fluid assessment with biweekly assessment of fetal growth. Management of MCTA complicated with sIUGR should be tailored to institutional resources and parents' wishes, and in our opinion, expectant management should be at least considered as a management option. An international registry of complicated MCTA triplets should be considered to help determine the best treatment option because of the paucity of cases.

## Data Availability

Data is available on request. You can contact the primary author via email laurence.carmant@umontreal.ca.
